# Fatigue, physical and daily activity, and self-perceived work ability in cancer survivors: a long-term follow-up study with a matched non-cancer reference group

**DOI:** 10.1007/s11764-026-02041-1

**Published:** 2026-05-21

**Authors:** Kristina Tödt, Maria Engström, Magnus Ekström, Marina Heiden, Angela G. E. M. de Boer, Anna Efverman

**Affiliations:** 1 Department of Caring Sciences, Faculty of Health and Occupational Studies, University of Gävle, Gävle, Sweden; 2 Respiratory Medicine, Allergology and Palliative Medicine, Department of Clinical Sciences Lund, Lund University, Lund, Sweden; 3 Department of Occupational Health, Psychology and Sports Sciences, Faculty of Health and Occupational Studies, University of Gävle, Gävle, Sweden; 4 Department of Public and Occupational Health, Amsterdam UMC - University of Amsterdam, Amsterdam, the Netherlands

**Keywords:** Fatigue, Cancer survivors, Daily activity, Physical activity, Work ability

## Abstract

**Purpose:**

To longitudinally assess fatigue, physical and daily activity, and work ability 7 years post therapy in a radiotherapy-treated cohort, and to compare the long-term data with matched non-cancer referents from the general population.

**Methods:**

A questionnaire assessed fatigue, concurrent symptoms, and physical and daily activity including work ability during radiotherapy and at a 7-year follow-up. For comparison, randomized matched population referents were included at the corresponding time point for each cancer survivor’s follow-up and responded the same questionnaire once. Generalized linear mixed models identified factors predicting and associated with fatigue and work ability at follow-up among survivors.

**Results:**

Of 221 cancer survivors (49% females; mean age 72 ± 11 years; 39% breast, 42% prostate, 20% other cancers) and 241 referents, survivors reported less fatigue over time but still more than referents (prevalence 58% vs. 41%, *P* = 0.001; score 2.1 vs. 1.3, *P* = 0.001). Their physical and daily activities improved to levels similar to referents. Working survivors reported lower self-perceived work ability (*P* = 0.016) and percentage of full-time work (*P* = 0.005). Fatigue during radiotherapy predicted fatigue at follow-up independent of depressed mood, pain, and number of concurrent symptoms. Among working survivors, fatigue was associated with poorer work ability, whereas being female, higher work self-efficacy, and work-related well-being were associated with better work ability.

**Conclusion and implication:**

Fatigue during treatment may be an early indicator for persistent fatigue in cancer survivors, which may be related to reduced work ability. Long-term cancer survivors reported worse fatigue compared with referents. Future studies are warranted to examine whether early identification and supportive strategies may influence later fatigue and its potential implications for work ability in cancer survivors.

**Supplementary Information:**

The online version contains supplementary material available at https://doi.org/10.1007/s11764-026-02041-1.

## Introduction

The number of cancer survivors is steadily increasing [1], many of whom live with persistent symptoms over many years [2]. Fatigue, defined as a pervasive, subjective sense of tiredness that persists over time, interfering with activities of daily living, and is not relieved by adequate rest [3], is one of the most common [4] and distressing [5] symptoms during cancer therapy. However, less is known about persistent fatigue in cancer survivors in the long term beyond 5 years of survivorship, especially in comparison with general population referents [6, 7].

Radiotherapy is part of approximately half of all cancer treatment regimens [8]. Nearly all patients experience some degree of fatigue during radiotherapy [4], and in a cross-sectional study of cancer survivors—predominantly 3 years post-treatment—73% reported experiencing fatigue at some point, with 57% experiencing fatigue on a daily basis [9].

Although fatigue is a well-known cancer-related symptom and effective evidence-based methods for managing fatigue exist [4], cancer survivors have described a lack of fatigue assessment and support for fatigue management [10, 11]. Persistent post-treatment fatigue has been associated with greater disability [12, 13], poorer quality of life [13], and greater health-care utilization [12] compared to survivors without fatigue. Depressed and anxious mood [13–15], comorbidity [7, 13, 16], type of cancer therapy, sociodemographic, and educational factors [7] have been reported to be associated with fatigue in long-term cancer survivors. Pre-treatment fatigue predicts fatigue up to 2.5 years after treatment [15, 16]. Longitudinal evidence indicates that fatigue remains relatively stable between 1 and 5 years post-treatment [17]. Moreover, fatigue assessed one year after diagnosis correlates significantly with fatigue measured 2 and 9 years later across multiple cancer types [18]. However, the extent to which fatigue during treatment predicts long-term fatigue remains unclear.

We previously reported that fatigue during radiotherapy was associated with lower levels of physical activity, greater limitations in daily activities, and poorer health-related quality of life [19]. More than two-thirds [20, 21] of various cancer populations do not adhere to physical activity guidelines [22], despite strong evidence showing that physical activity can alleviate fatigue and improve quality of life [23]. Rehabilitative interventions have been shown to reduce fatigue up to 10 years following cancer diagnosis [24]. There is a need for cancer care professionals to acquire knowledge that could inform earlier identification of cancer survivors at increased risk of long-term fatigue. Because most previous studies on fatigue in long-term cancer survivors have been cross-sectional [6, 7, 13, 14], there is a need to follow cancer survivors beyond 5 years of survivorship.

Persistent fatigue is associated with impaired work ability as has been observed in breast cancer survivors, evidenced in both short- and long-term follow-up studies [25, 26]. Factors influencing work ability include both health-related and work-related elements, such as the nature of the work and the availability of support and work adjustment options [27] and these factors may evolve over time [28]. Although cancer treatments, including chemotherapy, have been associated with an increased risk of long-term cancer survivors not returning to work [29], these immutable factors are of lesser concern to cancer care professionals who aim to identify modifiable factors that can be addressed through rehabilitation [22]. Consequently, factors that can be influenced, such as workplace adjustments and support, appear to be more critical when considering long-term work ability in cancer survivors [28, 30]. With the implementation of more flexible retirement policies and the gradual increase in the statutory retirement age, an increasing number of individuals remain in the workforce beyond the age of 67. This demographic shift underscores the need for research examining work ability [31] among older cancer survivors.

Fatigue is observed to decrease following treatment in the majority of cancer survivors [5]; however, there is limited understanding of the factors associated with fatigue several years after the completion of cancer treatment [11–14, 32], with few studies employing a longitudinal design [12, 32–34], or including a normative comparison group [6, 7]. The objective of this study was to longitudinally follow-up fatigue, physical and daily activity and work ability 7 years post therapy in a radiotherapy-treated cohort and to compare the long-term data with randomly selected matched non-cancer general population referents. Additionally, we investigated both prospective (factors assessed during radiotherapy, specifically fatigue) and cross-sectional factors (factors assessed at follow-up) associated with fatigue among cancer survivors. Among cancer survivors who were still working, we examined cross-sectional factors associated with self-perceived work ability.

## Methods

### Design and setting

In this longitudinal study, we conducted a follow-up of a cohort of cancer survivors approximately 7 years after receiving radiotherapy [19, 35], with or without adjunctive cancer therapies, at four oncology clinics located in central and southern Sweden. For comparison, a randomly selected matched non-cancer reference group from the general Swedish population was included at the corresponding time point for the cancer survivors’ follow-up. According to the Swedish social insurance system, physicians can prescribe sick leave in non-retired individuals in case of reduced working ability irrespective of whether the person is employed or not employed (job seekers, participating in job seeking, and job practice activities) or a student. The follow-up evaluation was conducted from May to December 2023.

### Inclusion of participants

We included cancer survivors who had undergone radiotherapy for cancer irrespective of cancer type or stage, who during their former radiotherapy were 18 years or older, and had participated in an earlier data collection [19, 35]. Cancer survivors were excluded if they exhibited physical, mental or language impairments—such as inability to communicate in Swedish—severe enough to preclude informed consent (e.g., dementia, terminal palliative cancer or other disease, or markedly impaired general condition). For each included cancer survivor, two individuals were randomly selected from the Swedish Tax Agency’s population registration database, matched by year of birth, sex, and geographic municipality. The same exclusion criteria applied to referents, with the additional criterion of no history registered-based of cancer or self-reported radiotherapy, at any point in life. Eligible cancer survivors and matched referents were invited to participate via mailed study information, with optional supplementary details provided orally or by email upon request, alongside a study questionnaire available in paper and online formats.

**Fig. 1:**
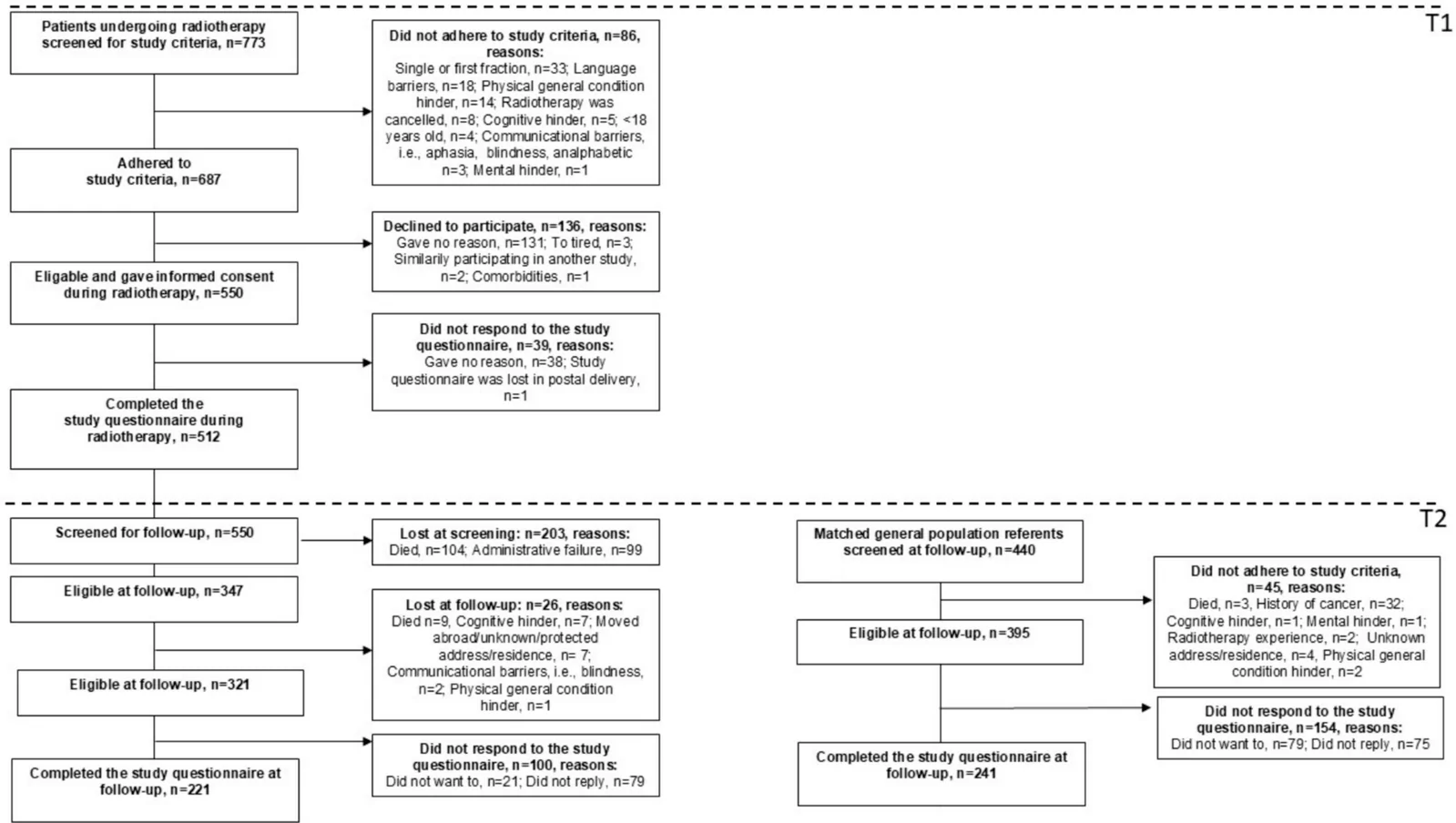
Flowchart of screened, included and responding participants (cancer survivors and referents). T1, number of cancer survivors when undergoing radiotherapy; T2, number of cancer survivors at 7-year follow-up

Of the 550 participants in the previous data collection [19, 35], 203 were lost during screening to follow-up (e.g., 104 had died). Of the remaining 347 eligible participants, 26 had barriers to participation that met the exclusion criteria or other reasons (Fig. 1). Of the remaining eligible 321, 100 did not respond to the questionnaire while 221 responded (69%). Of the 440 invited referents, 395 complied with study criteria while 45 did not, 154 did not respond to the study questionnaire, and 241 responded (61%) to the study questionnaire (Fig. 1).

### Data collection and procedures

#### Clinical data

For the cancer survivors, clinical data on cancer diagnosis, irradiated field, and dose of radiotherapy were extracted from medical records at the time of radiotherapy. All patients received external beam radiotherapy, as verified by the radiotherapy treatment records and confirmed by a radiotherapy nurse. Information on adjunctive cancer treatment beyond external radiotherapy was self-reported (yes/no) at both timepoints. The number of current diseases was calculated as the sum of self-reported affirmative responses (“yes, according to my physician”) by both cancer survivors and referents on item 3 of the Work Ability Index encompassing conditions such as musculoskeletal, cardiovascular, respiratory, mental, neurological, endocrine or metabolic diseases [36].

#### Questionnaire data

The study questionnaire employed in the present study was largely consistent with that employed in the previous radiotherapy data collection [19, 35]; however, radiotherapy-specific items were replaced with questions pertaining to lifestyle, and work-related issues. The referents received the same questionnaire as the cancer survivors but without cancer-specific questions. The study questionnaire began with demographic variables such as age, sex, marital status, education, occupation, and place of residence.

#### Fatigue and other symptoms

A clinimetrically developed [37] single-item measure assessed fatigue, “Have you felt an extreme tiredness that has been difficult to relieve by resting, during the past week?” (Supplemental Table S1). Responses were given on an 11-point scale (0 = never, not present and 1–10 = increasing frequency of fatigue with 10 = indicating constant fatigue) and are hereafter referred to as the fatigue score. This fatigue score was found to be highly correlated with other fatigue measures [19]. Pain intensity was evaluated using a numeric rating scale ranging from 0 = no pain to 10 worst imaginable pain. The Swedish version [38] of the Memorial Symptom Assessment Scale (MSAS) assessed the occurrence of 32 predefined symptoms.

#### Physical and daily activity, and work ability

Moderate and vigorous physical activities during the past week were assessed using seven and six fixed-response options, respectively [39]. The capacity to perform activities of daily living was assessed using a scale ranging from “I managed all of my daily activities” to “I did not manage any of my daily activities” [40] (Supplemental Table S1). Participants reported their current level of work or study as a percentage of full-time engagement (0–100%). Cancer survivors who were still working or studying at follow-up as well as working or studying referents rated their self-perceived work ability using the reliable and valid first item of the Work Ability Index [36, 41], previously applied in studies of cancer survivors [42]; on a scale from 0 (completely unable to work) to 10 (work ability at its best). At follow-up and as well the referents also rated their work performance compared to the performance of a “top worker”, a high-performing person with the same work as them (0: worst possible, to 10: best possible work performance) [43].

#### Other work-related issues among respondents who were still working

We used two clinimetrically developed items, based on previous research [36, 44], to assess work self-efficacy (0 = not at all confident to 10 = completely confident) and perceived work ability prior to cancer (0 = no ability to work to 10 = best possible work ability). One item assessed perceived flexibility granted by the employer in work execution on an 8-point scale (0 = not true at all, 7 = is completely true) [45]. Further, balance between work and leisure [46], feeling that the work is meaningful and “I feel good at work” (as an indicator of work-related well-being) [47], concerns about inability to perform work (0 = do not agree at all, to 7 = totally agree) [47], and level of support received (e.g., by the manager) in continuing work or returning to work (from 0 “no support” to 7 “best possible support”) were graded (Supplemental Table S1).

#### Perceived health and anxious and depressed mood

EQ-VAS (EuroQoL-Visual Analogue Scale) assessed overall health (0, worst imaginable health, to 100, best imaginable health) [48]. Psychological health in terms of anxious and depressed mood the past week was respectively rated from 0 = never to 7 = all the time [49].

### Statistical analysis

Descriptive statistics were reported as frequencies (*n*), percentages, mean (*m*) with ± standard deviation (SD). Chi-2, independent t-test and ANOVA were used to analyze differences in demographic and clinical characteristics, in fatigue change score, and current experience of fatigue between cancer survivors with different demographic and clinical characteristics. Using McNemar’s test, McNemar-Bowker test, Wilcoxon Signed Rank test, paired Student’s *t*-test (for nominal data, e.g., presence of fatigue yes/no; ordinal data, e.g., daily activity; and continuous data, e.g., fatigue score) respectively, we compared the cancer survivors at follow-up to when they were undergoing radiotherapy (dependent analyses), as well as to the matched referents (paired analyses). This analysis covered the descriptive variables seen in Table 1 (concurrent disease, health, anxious and depressed mood, and number of symptoms), fatigue score (score 0–10), physical and daily activity, self-perceived work ability, perceived work performance, and work in percentage of full-time work.

**Table 1:** Demographic and clinical characteristics, health and anxious and depressed mood, and number of symptoms in cancer survivors and matched referents

Variables	Cancer survivors *N* = 221	Referents *N* = 241	*P*-value (paired comparisons)
*Sociodemographic characteristics*			
Sex*,* *n (%)*			NA
Female	108 (48.9)	124 (51.5)	
Male	113 (51.1)	117 (48.5)	
Age, years, *m* (SD)	71.7 (11.1)	71.8 (10.4)	NA
Educational level*, n (%)*	*n* = *212*	*n* = *235*	0.430
Elementary	30 (14.2)	33 (14.0)	
Secondary	41 (19.3)	37 (15.7)	
Vocational	50 (23.6)	44 (18.7)	
University	91 (42.9)	121 (51.5)	
Marital status*, n (%)*	*n* = *218*	*n* = *237*	0.049
Married or co-habiting	157 (72.0)	175 (73.8)	
Living alone; have a partner	14 (6.4)	12 (5.1)	
Living alone; single or widow/widower	47 (21.6)	50 (21.1)	
Place of residence*, n (%)*	*n* = *214*	*n* = *226*	0.002
Big town	104 (48.6)	138 (61.1)	
Mid-size town	33 (15.4)	47 (20.8)	
Small town	31 (14.5)	19 (8.4)	
Rural area	46 (21.5)	22 (9.7)	
Main occupational status*, n (%)*	*n* = *216*	*n* = *236*	NA*
Employed^a^	51 (23.6)	53 (22.5)	
On sick leave	1 (0.5)	1 (0.4)	
Retired^b^	157 (72.7)	181 (76.7)	
Not employed, student, other	5 (2.3)	1 (0.4)	
Disability pension	2 (0.9)	0	
*Clinical characteristics*		NA	
Cancer type*, n (%)*			
Breast	85 (38.5)		
Prostate	92 (41.6)		
Head/neck and brain	13 (5.9)		
Colon, rectal, anal, and gynaecological	20 (9.0)		
Other cancers, e.g., lung, lymphoma, cancer in connective tissues	11 (5.0)		
Extensive disease*, n (%),* *n* = 192	25 (13.0)		
Cancer therapies received at any time,* n (%)*			
External radiotherapy, *n* = *221*	221 (100)		
Internal radiotherapy, *n* = *215*	47 (21.9)		
Tumour surgery,* n* = *216*	154 (71.3)		
Chemotherapy, *n* = *216*	81 (37.5)		
Hormonal therapy,* n* = *213*	97 (45.5)		
Received any form of cancer rehabilitation, *n* = *188*	33 (17.6)		
Concurrent diseases^c^	*n* = *212*	*n* = *229*	
Number of diseases, m (SD)	1.6 (1.6)	1.3 (1.3)	0.008
Comorbidity ≥ 2 diseases, *n* (%)	95 (45)	79 (33)	0.067
BMI, m (SD),	*n* = *205* 25.8 (3.7)	*n* = *227* 25.2 (3.9)	0.618
*Health, anxious and depressed mood, and symptoms*			
EQ-VAS^d^ (0–100), m (SD)	*n* = *208* 76.1 (18.1)	*n* = *221* 78.3 (19.6)	0.165
Anxious mood, m (SD)	*n* = *212* 0.7 (1.3)	*n* = *225* 0.5 (1.1)	0.081
Depressed mood, m (SD)	*n* = *213* 1.1 (1.6)	*n* = *226* 0.7 (1.3)	0.067
Number of concurrent symptoms^e^, m (SD)	*n* = *211* 8.5 (7.8)	*n* = *226* 5.7 (6.2)	< 0.001

Data are presented as frequency (*n*) with percentage. The number of participants providing data is indicated in cases of missing data. Abbreviations: *NA*, not applicable, referents were matched according to sex and year of birth; NA*, not applicable due low *n*; *m*, mean; *SD*, standard deviation; *BMI*, body mass index. ^a^Employed and self-employed. ^b^Include 16 partly retired cancer survivors and 22 partly retired referents. ^c^Sum of self-reported affirmative responses 0–13 (“yes, according to my physician”) on item 3 of the Work Ability Index (tumour or cancer excluded) *n* of cancer survivors/referents responding yes on respective disease: Injury due to an accident *n* = 11/*n* = 14, musculoskeletal disease *n* = 32/*n* = 37, cardiovascular disease *n* = 77/*n* = 85, respiratory disease *n* = 21/*n* = 24, mental disorder *n* = 12/*n* = 7, neurological or sensory disease *n* = 27/*n* = 21, digestive disease/condition *n* = 13/*n* = 17, genitourinary disease *n* = 29/*n* = 16, skin disease *n* = 10/*n* = 18, endocrine or metabolic disease *n* = 34/*n* = 22, blood disease *n* = 4/*n* = 2, birth defect *n* = 1/*n* = 1, other disease *n* = 16/*n* = 20 [36]. ^d^*EQ-VAS*, EuroQol Visual Analogue Scale ranging from 0 to 100, where higher scores indicate better perceived health status. ^e^Total number of symptoms present based on the occurrence of up to 32 possible symptoms as assessed by the Memorial Symptom Assessment Scale [38].

For the cancer survivors, fatigue change scores were classified as follows: a change greater than ± 10%, i.e., ± 1 point was considered significant, with change scores from −1 to 1 indicating stable (unchanged) fatigue, scores from −2 to −10 indicating decreased fatigue, and scores from + 2 to + 10 indicating worsened fatigue based on previous findings of a minimal important difference on the 0–10 visual analogue scale for fatigue which has been reported to range from 0.8 to 1.1 for improvement and from 1.0 to 1.3 for worsening [50]. We used generalized linear mixed models (GLMM) to determine whether factors assessed during the previous radiotherapy (prospective factors) predicted fatigue (score 0–10) at follow-up, Model 1. In Model 2, we analyzed if fatigue score during the previous radiotherapy still predicted fatigue score at follow-up, after adding variables assessed at follow-up (cross-sectional factors). Models 1–2 were adjusted for age, sex, cancer type, and treatment with chemotherapy at any time (yes/no), and independent variables were selected based on existing literature [7, 12–16, 19] and informed by the Theory of Unpleasant Symptoms [51]: anxious and depressed mood (0 = never to 7 = all the time), comorbidity at the time of radiotherapy (during) and concurrent diseases at follow-up (yes/no), number of symptoms (0–32 possible number of symptoms according to MSAS, excluding lack of energy), pain, and the lifestyle physical activity (moderate physical activity < versus > 150 min/week). In a third model, Model 3, we analyzed factors at follow-up (cross-sectional factors) associated with self-perceived work ability (score 0–10). For this GLMM, only full-time retired cancer survivors who were not working at all were excluded, i.e., employed, self-employed, students and partly working retired survivors were included in the analysis. Independent variables comprised those factors that demonstrated the strongest correlation with work ability [52], and adjusted for age and sex. In all GLMM models, listwise deletion was used. Site was entered as a random effect and remaining factors as fixed effects. Variance components were used as the covariance structure, as it provided better model fit according to AIC and BIC compared with an unstructured covariance type. Inspection of the residuals in the GLMM models revealed no harmful deviation from normality (skewness: −0.19 to 1.30; kurtosis: 0.38 to 2.82). Response analyses were conducted to verify that data loss in the GLMM models did not affect their results. Statistical significance was defined as a two-tailed *P* < 0.05. All analyses were performed in IBM SPSS statistics version 27.0 (IBM Corp, Armonk NY, USA).

## Results

### Participants

The 221 cancer survivors had a mean age of 71.7 ± 11.1 years, 108 (49%) were female, and 18% recalled having received cancer rehabilitation at some point (Table 1). Compared with responders, non-responders (*n* = 100) were younger (66.8 ± 13.5 years) and had a higher proportion of females (73%). There was no significant difference in radiotherapy dose between responders and non-responders, with mean dose of 56.4 ± 14.3 and 52.3 ± 12.5 Gy, respectively. The cancer survivors had previously been treated for breast cancer (39%), prostate cancer (42%), or other cancer types (20%). The referents had demographic characteristics similar to those of the cancer survivors, although a greater proportion resided in urban areas. The majority of both cancer survivors and referents (> 70%) were married or cohabiting, and 43% of cancer survivors and 52% of referents had attained a university-level education. Of the 201 cancer survivors who responded to this self-report question, 58 (29%) reported having a cancer diagnosis at follow-up. At follow-up, an additional 17 cancer survivors reported having received chemotherapy compared with during radiotherapy, 23 reported having undergone internal radiotherapy, 22 received antihormonal therapy, and 34 had undergone tumour surgery.

### Fatigue at follow-up compared to while undergoing radiotherapy and compared to referents

Of the 221 cancer survivors, 211 (95%) reported fatigue data. No fatigue was reported by 89 (42%); fatigue score 1–3 was reported by 71 (34%), score 4–6 by 29 (14%), and score 7–10 by 22 (10%). The mean change in fatigue score (*n* = 195 delivered fatigue data at both time points) was −0.6 (95% CI −0.9 to −0.1) (Table 2). Based on a change score of ± ≥ 10%, 68 (35%) were classified as alleviated, 97 (50%) as unchanged, and 30 (15%) as worsened.

**Table 2:** Changes in fatigue from during previous radiotherapy to follow up in cancer survivors with varying sociodemographic, clinical, and life-style factors and factors predicting and associated with fatigue at follow-up

	Change in fatigue score *m* difference (95% *CI*)	*P*-value, subgroup comparison change score	Fatigue score at follow-up, *m* (95% *CI*)	*P*-value, subgroup comparison at follow-up	GLMM Model 1, *n* = 179		GLMM Model 2 *n* = 179	
					Beta (95% CI)	*P*-value	Beta (95% CI)	*P*-value
Cancer survivors with fatigue scores during and at follow-up, *n* = *195*	−0.6 (−0.9 to −0.1)	0.010	2.1 (1.7 to 2.5)					
Variables								
*Sociodemographic factors*								
Age^a^		0.139		0.296	0.009 (−0.029 to 0.048)	0.638	0.013 (−0.023 to 0.049)	0.485
18–44 years, *n* = 13	−0.3 (−2.7 to 2.1)		3.2 (1.0 to 5.5)					
45–69 years, *n* = 110	−0.9 (−1.5 to −0.4)		2.0 (1.5 to 2.6)					
≥ 70 years, *n* = 72	0.0 (−0.8 to 0.7)		2.0 (1.5 to 2.6)					
Sex		0.333		0.011				
Male, *n* = 100	−0.4 (−0.9 to 0.2)		1.6 (1.1 to 2.1)		Ref		Ref	
Female, *n* = 95	−0.8 (−1.4 to −0.2)		2.6 (2.0 to 3.2)		0.544 (−0.954 to 2.043)	0.474	−0.035 (−1.322 to 1.253)	0.958
Marital status		0.314		0.407	-		-	
Cohabiting, having a partner, *n* = 151	−0.5 (−1.0 to 0.0)		2.0 (1.6 to 2.5)					
Single, without a partner, *n* = 43	−1.0 (−1.8 to −0.2)		2.4 (1.6 to 3.2)					
Educational level		0.298		0.778	-		-	
Elementary, *n* = 28	0.1 (−1.2 to 1.4)		2.3 (1.3 to 3.4)					
Secondary and vocational, *n* = 86	−0.5 (−1.2 to 0.2)		2.0 (1.4 to 2.5)					
University, *n* = 76	−0.9 (−1.5 to −0.3)		2.2 (1.6 to 2.8)					
*Clinical factors*								
Cancer type,		0.777		0.034				
Breast cancer, *n* = 74	−0.6 (−1.3 to 0.2)		2.6 (2.0 to 3.3)		Ref		Ref	
Prostate cancer, *n* = 80	−0.4 (−1.0 to 0.3)		1.5 (1.1 to 2.0)		−0.270 (−1.930 to 1.391)	0.749	−0.287 (−1.732 to 1.158)	0.695
Colon, rectal, anal, and gynaecological cancer, *n* = 18	−1.3 (−2.6 to 0.1)		1.6 (0.6 to 2.6)		−1.143 (−2.584 to 0.298)	0.119	−0.926 (−2.250 to 0.398)	0.169
Head/neck and brain cancer,* n* = 12	−0.9 (−2.7 to 0.9)		2.0 (0.1 to 3.9)		−0.453 (−2.390 to 1.483)	0.645	0.159 (−1.411 to 1.730)	0.841
Other cancers, e.g., lung, lymphoma, cancer in connective tissues, *n* = 11	0.4 (−2.7 to 2.0)		3.4 (0.9 to 5.8)		0.615 (−1.129 to 2.360)	0.487	0.031 (−1.493 to 1.555)	0.968
Chemotherapy^b^		0.422		0.107				
Yes, *n* = 75	−0.8 (−1.4 to −0.0)		2.5 (1.8 to 3.2)		0.222 (−0.752 to 1.197)	0.653	0.218 (−0.651 to 1.087)	0.621
No, *n* = 119	−0.4 (−0.9 to 0.0)		1.9 (1.4 to 2.3)		Ref		Ref	
Hormonal therapy^b^		0.458		0.372	-		-	
Yes, *n* = 88	−0.8 (−1.4 to −0.1)		2.3 (1.7 to 2.9)					
No, *n* = 104	−0.4 (−1.0 to 0.2)		2.0 (1.4 to 2.5)					
Internal radiotherapy^b^		0.573		0.761	-		-	
Yes, *n* = 44	−0.3 (−1.1 to 0.5)		2.2 (1.5 to 3.0)					
No,* n* = 150	−0.6 (−1.1 to −0.1)		2.1 (1.6 to 2.5)					
Comorbidity during previous radiotherapy		0.354		0.012			-	
Yes, *n* = 76	−0.8 (−1.5 to −0.0)		2.7 (2.0 to 3.4)		0.188 (−0.639 to 1.014)	0.655		
No, *n* = 109	−0.3 (−0.9 to 0.2)		1.7 (1.2 to 2.1)		Ref			
Concurrent diseases at follow-up, 0–13^c^		0.989		0.174			−0.092 (−0.318 to 0.133)	0.419
Yes, *n* = 137	−0.6 (−1.1 to −0.1)		2.3 (1.8 to 2.7)					
No, *n* = 54	−0.6 (−1.4 to 0.3)		1.7 (1.0 to 2.4)					
Cancer rehabilitation		0.990		0.199			-	
Yes, *n* = 31	−0.6 (−1.6 to 0.4)		2.7 (1.6 to 3.8)					
No, *n* = 136	−0.6 (−1.1 to −0.1)		1.9 (1.5 to 2.4)					
*Health, anxious, and depressed mood*								
* During previous radiotherapy*								
Depressed mood, 0–7^d^		0.012		0.001	−0.118 (−0.484 to 0.248)	0.526		
Yes (1–7), *n* = 100	−1.1 (−1.7 to −0.5)		2.7 (2.2 to 3.3)					
No (0), *n* = 94	0.0 (−0.6 to 0.6)		1.5 (1.0 to 2.0)					
Anxious mood, 0–7^d^		0.018		0.001	0.307 (−0.076 to 0.690)	0.115		
Yes (1–7), *n* = 61	−1.4 (−2.3 to −0.5)		3.2 (2.4 to 4.0)					
No (0), *n* = 133	−0.2 (−0.6 to 0.3)		1.6 (1.2 to 2.0)					
*At follow-up*								
Depressed mood, 0–7^d^		0.119		< 0.001	-		0.545 (0.281 to 0.809)	< 0.001
Yes, *n* = 89	−0.2 (−0.9 to 0.5)		3.3 (2.7 to 3.9)					
No, *n* = 104	−0.9 (−1.4 to −0.4)		1.1 (0.7 to 1.5)					
Anxious mood, 0–7^d^		0.889		< 0.001	-		0.105 (−0.223 to 0.433)	0.529
Yes (1–7), *n* = 56	−0.5 (−1.4 to 0.4)		3.3 (2.4 to 4.1)					
No (0), *n* = 136	−0.6 (−1.1 to −0.1)		1.7 (1.3 to 2.1)					
*Symptoms*								
*During previous radiotherapy*								
Fatigue during^e^, 0–10		< 0.001		< 0.001	0.294 (0.113 to 0.474)	0.002	0.157 (0.027 to 0.286)	0.018
No fatigue 0, *n* = 60	1.2 (0.6 to 1.8)		1.2 (0.6 to 1.8)					
Fatigue 1–3, *n* = 74	−0.3 (−0.8 to 0.2)		1.7 (1.2 to 2.2)					
Fatigue 4–6, *n* = 34	−1.5 (−2.7 to −0.3)		3.1 (2.0 to 4.3)					
Fatigue 7–10, *n* = 27	−4.0 (−4.9 to −3.0)		4.1 (3.0 to 5.2)					
Number of symptoms^f^, 0–31^f^		0.011		< 0.001	0.078 (0.008 to 0.149)	0.029		
3 symptoms or less, *n* = 59	0.1 (−0.5 to 0.7)		1.2 (0.7 to 1.7)					
4 symptoms or more, *n* = 133	−0.8 (−1.4 to −0.3)		2.5 (2.0 to 3.0)					
Pain^g^, 0–10		0.005		0.027	−0.097 (−0.317 to 0.123)	0.385		
Pain (1–10), *n* = 91	−1.2 (−1.9 to −0.5)		2.6 (2.0 to 3.2)					
No pain (0), *n* = 103	0.0 (−0.5 to 0.5)		1.7 (1.2 to 2.2)					
*At follow-up*								
Number of symptoms^f^, 0–31^f^		0.595		< 0.001			0.085 (0.036 to 0.134)	< 0.001
2 symptoms or less, *n* = 54	−0.7 (−1.4 to −0.0)		0.9 (0.4 to 1.4)					
3 symptoms or more, *n* = 137	−0.5 (−1.0 to 0.1)		2.6 (2.1 to 3 0.1)					
Pain^g^, 0–10		0.009		< 0.001			0.144 (0.010 to 0.277)	0.035
Pain (1–10), *n* = 116	−0.1 (−0.7 to 0.5)		3.0 (2.5 to 3.6)					
No pain (0), *n* = 78	−1.2 (−1.8 to −0.7)		0.8 (0.4 to 1.1)					
*Lifestyle factors*								
Moderate physical activity *during previous radiotherapy*		0.071		0.862				
0–150 min, *n* = 135	−0.8 (−1.3 to −0.3)		2.1 (1.7 to 2.6)					
> 150 min, *n* = 59	0.0 (−0.8 to 0.9)		2.1 (1.4 to 2.7)					
Moderate physical activity *at follow-up*		0.748		0.016				
0–150 min, *n* = 111	−0.5 (−1.1 to 0.1)		2.5 (1.9 to 3.1)				Ref	
> 150 min, *n* = 79	−0.7 (−1.3 to −0.0)		1.6 (1.1 to 2.1)				−0.228 (−0.903 to 0.448)	0.507
Body mass index *at follow-up*		0.397		0.528			-	
< 25, *n* = 83	−0.8 (−1. 4 to −0.2)		2.3 (1.7 to 2.9)					
≥ 25, *n* = 101	−0.4 (−1.1 to 0.2)		2.1 (1.5 to 2.6)					
Smoking *at follow-up*		0.585		0.303			-	
Current smoker,* n* = 12	− 0.1 (−1.8 to 1.7)		2.9 (0.6 to 5.2)					
Never and former smoker,* n* = 180	−0.6 (−1.0 to −0.1)		2.1 (1.7 to 2.5)					

*P*-values represent comparisons between cancer survivors stratified by different characteristics. The number of participants (*n*) providing data is indicated in cases of missing data. m, mean; SD, standard deviation. Change score refers to the difference in fatigue score between radiotherapy and follow-up. ^a^Age groups are based on Statistics Sweden’s population classifications; ^b^Treated with chemotherapy and/or hormonal therapy at any time. ^c^Concurrent diseases possible range 0–13, yes = one or more diseases according to item 3 of the Work Ability Index (tumour or cancer excluded) [36]; ^d^Depressed mood or anxious mood, no = 0, never, and yes = presence of any depressed mood or anxiety in the past week, score 1–7, with 7 = all the time; ^e^fatigue; 0 = never, no fatigue, 1 to 10 = all the time, ^f^Total number of symptoms present based on the occurrence of up to 31 possible symptoms as assessed by the Memorial Symptom Assessment Scale (excluding lack of energy) [38], ^g^pain; 0 = no pain, 1 to 10 = worst pain imaginable, ^f^total number of symptoms present categorized as ≤ 3 symptoms versus > 3 symptoms during previous treatment and current number ≤ 2 versus > 2 (cut off at the 25th percentile)

**Fig. 2:**
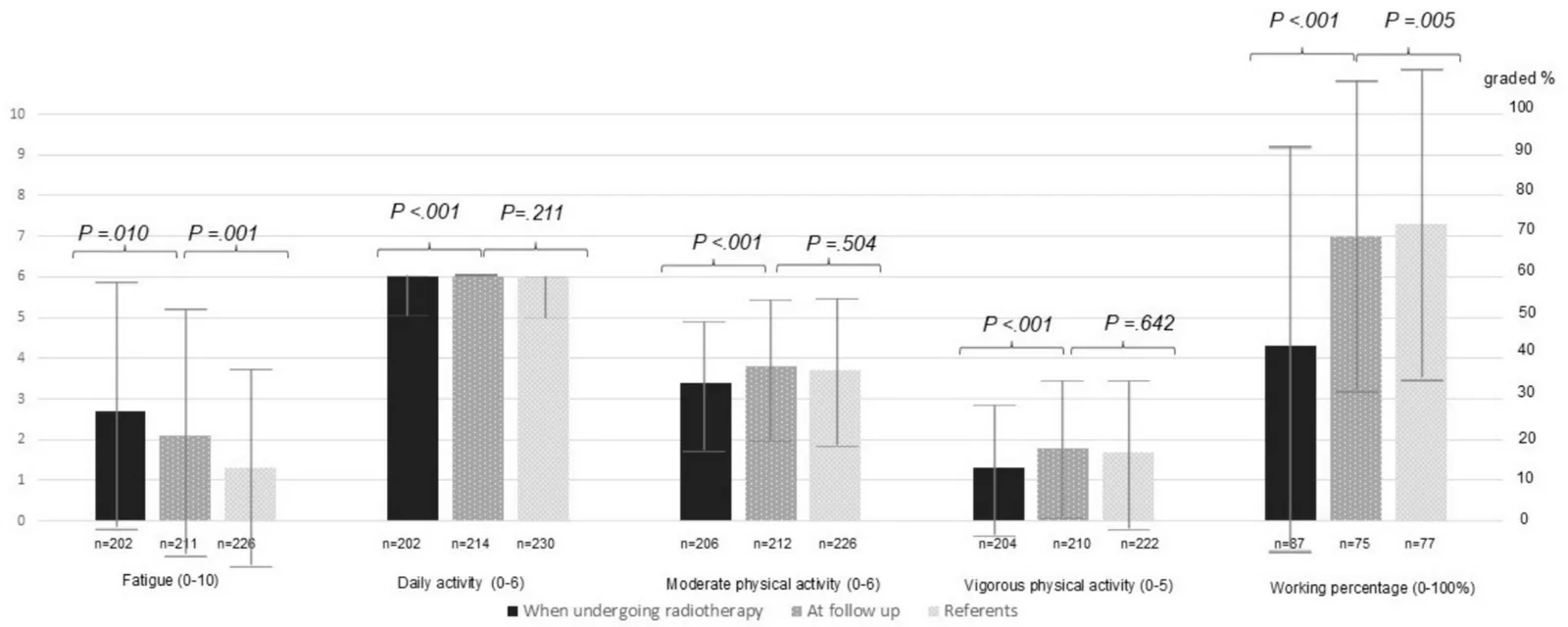
Fatigue (0 = no fatigue to 10 = fatigue all the time), daily activity (0 = have not managed any of the daily activities to 6 = managed all daily activities), physical activity (moderate physical activity 0 = no minutes the past week to 6 = > 300 min, vigorous physical activity 0 = no minutes the past week to 5 = > 120 min), and work in percentage of full-time work, when undergoing radiotherapy and at the long-term follow-up, compared to the referents. *m* value and ± 1 standard deviation are presented for the numeric rating scales. *Md* values and 25th and 75th percentile are presented for the ordinal category rating scale, i.e., daily activity. *P*-values regard student’s paired t-test and comparisons in daily activity using the Wilcoxon Signed-rank Test

Paired analysis revealed a significantly lower proportion reporting fatigue at follow-up (58% vs. 69%, *P* = 0.005), as well as lower mean fatigue score (2.1 ± 2.7 vs. 2.7 ± 2.8; *P* = 0.010) (Fig. 2). However, both the prevalence and fatigue mean score at follow-up were higher than among referents; prevalence (58% vs. 41%, *P* = 0.001) and score (2.1 ± 2.7 vs. 1.3 ± 2.2; *P* = 0.001) (Fig. 2).

### Prospective and cross-sectional factors associated with fatigue at follow-up

Bivariate analyses of fatigue change score demonstrated significantly greater reduction in fatigue score among cancer survivors reporting depressed mood, anxiety, 4 or more concurrent symptoms, and pain during the previous radiotherapy as well as among those exhibiting higher fatigue score during the previous radiotherapy (Table 2).

In the bivariate analyses, higher fatigue scores at follow-up were significantly associated with female sex, presence of comorbidity and anxious and depressed mood, higher fatigue scores, greater number of concurrent symptoms and pain reported during the previous radiotherapy as well as with anxious and depressed mood and a greater number of concurrent symptoms and pain reported at follow-up. Reporting ≤ 150 min of moderate physical activity per week at follow-up was also significantly associated with higher fatigue score. According to Model 1 (*n* = 179 included in the analysis), fatigue and number of concurrent symptoms experienced during the previous radiotherapy were independent, significant predictors of fatigue at follow-up (Table 2). In Model 2 (*n* = 179), analyzing fatigue score during radiotherapy together with cross-sectional factors associated with fatigue at follow-up, fatigue during radiotherapy remained a significant predictor. Additionally current depressed mood, pain, and number of the concurrent symptoms remained independently associated with fatigue (Table 2).

### Physical and daily activity at follow-up compared to while undergoing radiotherapy

Moderate and vigorous physical activity, as well as the ability to manage daily activities, improved at follow-up (all *P* < 0.001) (Fig. 2). The proportion of practicing at least 150 min of moderate physical activity during the past week was 40% among cancer survivors and 41% among referents (*P* = 1.000). Most cancer survivors and the referents managed all their daily activities 163 (76%) and 190 (83%), respectively (*P* = 0.211).

### Work ability

Engagement in occupational work or study to at least some extent was reported by 75 (35%, *n* = 75/216) cancer survivors and 77 (33%, *n* = 77/236) referents (*P* = 0.568). At follow-up, cancer survivors reported to work equivalent to 69.6 ± 35.2% of full-time engagement, which was significantly higher than during radiotherapy, 42.5 ± 46.6%, *P* < 0.001, yet was slightly lower than that of referents 73.0 ± 34.8%, *P* = 0.005 (Fig. 2). Approximately one in ten of the cancer survivors (*n* = 21/203, 10%) and referents (*n* = 18/223, 8%) had retired earlier than expected, and n = 14 of 203, 7% of the cancer survivors and *n* = 14 of 223, 6% of the referents had changed employment due to health issues.

Among cancer survivors (*n* = 69), the mean self-perceived work ability was 7.9 ± 2.2, compared with 8.0 ± 2.2 among referents (*n* = 73) (*P* = 0.016). The mean rated work performance was 8.4 ± 1.7 among cancer survivors (*n* = 65) and 8.5 ± 1.6 among referents (*n* = 68) (*P* = 0.037). Additionally, cancer survivors rated their current work ability as significantly lower than their work ability prior to cancer diagnosis (mean 9.1 ± 1.9, *P* < 0.001).

### Factors associated with self-perceived work ability

Age, sex, educational level, cancer type or prior chemotherapy were not significantly associated with cancer survivors’ self-perceived work ability in bivariate analyses. Current fatigue (*r* = −0.660) rated work ability prior to cancer (*r* = 0.648) and work self-efficacy (*r* = 0.589) showed the strongest correlations with self-perceived work ability; these and other significantly correlating factors are presented in Table 3. In GLMM 3 (*n* = 52 included in the analysis) being female, current fatigue, work self-efficacy, and work-related well-being were identified to be significantly and independently associated with self-perceived work ability (Table 3).

**Table 3:** Factors associated with self-perceived work ability in cancer survivors at long-term follow-up

Working cancer survivors *n* = 75	Self-perceived work ability^a^, *m* (SD)	*P*-value, subgroup comparison	Pearson Correlation coefficient, *r*	*P*-value, correlation coefficient	GLMM Model 3 *n* = 52 (female, *n* = 38, male, *n* = 14)	
					Beta (95% CI)	*P*-value
Self-perceived work ability^a^, *n* = 69	7.9 (2.2)					
*Sociodemographic factors*						
Age, *n* = 69		0.601	−0.040	0.745	−0.013 (−0.052 to 0.025)	0.487
< 65 years, *n* = 45	8.0 (2.1)					
≥ 65 years, *n* = 24	7.7 (2.4)					
Sex, *n* = 69		0.163				
Male, *n* = 19	7.3 (2.6)				Ref	
Female, *n* = 50	8.1 (2.0)				1.008 (0.052 to 1.964)	0.039
Educational level, *n* = 68		0.319				
Elementary, *n* = 1	10					
Secondary and vocational, *n* = 30	8.2 (2.3)					
University, *n* = 37	7.7 (1.7)					
*Clinical factors*						
Cancer type,* n* = 68		0.203				
Breast, *n* = 39	8.2 (2.1)					
Prostate, *n* = 12	7.8 (1.7)					
Colon, rectal, anal and gynaecological,* n* = 7	7.9 (1.5)					
Head/neck and brain,* n* = 9	6.4 (3.1)					
Lung, lymphoma and cancer in supportive tissues, *n* = 2	6.5 (0.7)					
Chemotherapy^b^, *n* = 69		0.995				
Yes, *n* = 41	7.9 (2.1)					
No, *n* = 28	7.9 (2.2)					
Concurrent diseases^c^ at follow-up, (0–13), *n* = 67			−0.258	0.035		
*Lifestyle factors*						
Body Mass Index, *n* = 66			−0.079	0.528		
Moderate physical activity past week at follow-up,* n* = 69		0.659				
0–150 min, *n* = 42	7.8 (2.2)					
> 150 min, *n* = 27	8.0 (2.1)					
*Work-related factors*						
Ability to work preceding the cancer^d^ (0–10), *n* = 67			0.648	< 0.001	0.137 (−0.228 to 0.502)	0.452
Work self-efficacy^e^ (0–10); *n* = 67			0.589	< 0.001	0.331 (0.050 to 0.613)	0.022
My work is meaningful^f^ (0–7),* n* = 68			0.283	0.019		
Work-related well-being^f^ (0–7), *n* = 68			0.396	< 0.001	0.315 (0.003 to 0.628)	0.048
I have a good balance between work and leisure^f^ (0–7), *n* = 68			0.370	0.002	−0.104 (−0.325 to 0.117)	0.347
Worries about the ability to perform the work^f^ (0–7), *n* = 68			−0.325	0.007	−0.197 (−0.395 to 0.000)	0.051
Flexibility at work given by manager^g^ (0–7), *n* = 50			−0.170	0.237		
Perceived support from manager^h^ (0–7), *n* = 56			−0.088	0.521		
Perceived support from colleges/co−workers^h^ (0–7),* n* = 59			−0.062	0.641		
Perceived support from family and friends^h^ (0–7), *n* = 60			0.058	0.659		
*Health, anxious and depressed mood, and symptoms*						
EQ-VAS^i^ (0–100), *n* = 69			0.169	0.164		
Fatigue score at follow-up^j^ (0–10), *n* = 69			−0.660	< 0.001	−0.211 (−0.327 to −0.094)	< 0.001
Pain^k^ (0–10), *n* = 69			−0.223	0.065		
Anxiety^l^ (0–7),* n* = 69			−0.235	0.052		
Depressed mood^l^ (0–7), *n* = 69			−0.146	0.230		
Number of concurrent symptoms^m^ (0–32), *n* = 67			−0.467	< 0.001	−0.045 (−0.103 to 0.012)	0.120

Number, *n*, of participants delivering data is presented in case of missing data. Full-time retired cancer survivors who were not working at all were excluded. *m*, mean; *SD*, standard deviation is presented for categorical comparisons and Pearson correlation coefficient (*r*) for continuous data is presented. Statistically significant estimates are shown in bold. ^a^Self-perceived work ability; 0 = can’t work at all to 10 = ability to work is at its best, ^b^Treated with chemotherapy at any time,^c^Sum of self-reported affirmative responses 0–13 (“yes, according to my physician”) on item 3 of the Work Ability Index (tumour or cancer excluded) [36], ^d^Work ability preceding cancer; 0 = no work ability to 10 = best possible work ability, ^e^Work self-efficacy; 0 = not confident to 10 = completely confident, ^f^“My work is meaningful”, Work-related well-being, “I have a good balance…”, “Worries about the ability…”; 0 = don’t agree to 7 = agree completely, ^g^Flexibility at work, 0 = not true at all to 7 completely true, ^h^Perceived support; 0 = no support to 7 best possible support, ^i^EQ-VAS, EuroQoL-Visual Analogue Scale; 0–100, higher values the better perceived health. ^j^Fatigue; 0 = never to 10 = all the time, ^k^Pain; 0 = no pain, 1 to10 = worst pain imaginable, ^l^Anxiety and depressed mood; 0 = never to 7 = all the time., ^m^ Total number of symptoms present based on the occurrence of up to 32 possible symptoms as assessed by the Memorial Symptom Assessment Scale [38]

## Discussion

### Main findings

The main findings of this study indicate that both prevalence and frequency of fatigue decreased significantly over 7-year follow-up in post-therapy cancer survivors, but were higher than in population referents. Levels of physical activity and ability to manage daily activities showed significant improvement to follow-up and matched those of the referents. However, cancer survivors who were working at follow-up reported lower self-perceived work ability than referents. Fatigue experienced during radiotherapy predicted fatigue at follow-up, independent of depressed mood, pain, and number of concurrent symptoms. Among working cancer survivors, current fatigue was associated with poorer self-perceived work ability. In contrast, being female, better work self-efficacy and work-related well-being were associated with greater self-perceived work ability.

### Interpretation

Although fatigue decreased over time, more than half of survivors reported fatigue at follow-up, with scores remaining stable (change −1 to 1) or even worsening in some compared to when undergoing radiotherapy. This is concerning, particularly as only 18% recalled receiving cancer rehabilitation. Nearly one quarter experienced severe, non-relievable fatigue almost half of the time or more, likely substantially impairing daily functioning. Fatigue is among the most burdensome long-term sequelae of cancer [11]. Our findings are consistent with prior studies, including a cohort of over 4000 non-Hodgkin lymphoma survivors in which 56–64% reported persistent fatigue 5 years post-treatment [33]. In our sample, approximately one quarter (51/211) reported fatigue scores ≥ 4, comparable to 29% (115/515) reporting ≥ 4 on a fatigue intensity scale 0–10 in another survivor cohort of various cancers 5 years after treatment [53], and similar to reports that one in three survivors experience clinically significant fatigue affecting daily life [34]. Although earlier studies lacked general population comparator, both our findings and previous research indicate higher fatigue level among cancer survivors than non-cancer referents [6, 7]. Together, these results underscore the substantial prevalence of long-term fatigue in this population [2].

Fatigue experienced during radiotherapy predicted fatigue 7 years post treatment, underscoring the importance of systematic fatigue assessment and management throughout cancer care. Previous research has identified pretreatment fatigue as a predictor of post-treatment fatigue [15, 16], also following cancer rehabilitation [54]. Our result suggests that the timing of the assessment before treatment initiation may be less critical than ensuring that it is conducted. Although several studies, including ours, have reported associations between fatigue and depressed and anxious mood [13–15], findings are inconsistent [7, 12, 16]. Comorbidity was associated with fatigue in bivariate analyses, consistent with prior research [7, 16]; however, this association did not remain significant in the adjusted model. Among people with various chronic diseases, the proportion experiencing severe fatigue increased with the number of chronic diseases [55]. However, our result suggests that it is the experience of concurrent symptoms that is more important, and not the presence of any comorbidity per se. Screening for cancer-related distress may facilitate identification of rehabilitation needs and appropriate supportive interventions [56, 57]. Notably, only 18% of the survivors reported having received any cancer rehabilitation in the present study, which may highlight a persistent gap in care [58]. In contrast to a cross-sectional study [7], age, sex and cancer type did not predict long-term fatigue in our study. Together, these results emphasize that symptoms experienced during active treatment, rather than treatment modality alone, may shape long-term survivorship outcomes, as also observed in Hodgkin’s lymphoma survivors [33].

Fortunately, cancer survivors did not report poorer capacity to perform daily activities or engage in moderate-to-vigorous physical activity than referents, despite experiencing worse fatigue and concurrent symptoms. This is encouraging and may reflect successful implementation of evidence supporting physical activity as a strategy to alleviate fatigue in routine cancer care [22]. Alternatively, a response shift [59] may have occurred, whereby survivors recalibrate their expectations and adapt to a “new normal”. Qualitative research indicates that many cancer survivors learn to accept and accommodate persistent fatigue [60], which may represent an unmet supportive care need. This “new normal” due to fatigue was also identified in oropharyngeal cancer survivors being interviewed 2 to 9 years after radiotherapy [61]. Similarly, the absence of lower EQ-VAS ratings among cancer survivors compared with referents may reflect such recalibration [59], and should be considered in rehabilitation screening.

Cancer survivors reported lower self-perceived work ability and lower work participation than referents consistent with findings by Le et al. [62] demonstrating reduced employment in cancer survivors compared to non-cancer individuals based on registry income data. These converged findings suggest a need for work-oriented rehabilitation throughout long-term survivorship. In a previous study, we found that depressed mood explained more variance in sickness absence during radiotherapy than fatigue [19]. However, 7 years after radiotherapy—consistent with earlier studies [27, 42]—fatigue emerged—independent of depressed mood to be an important symptom for healthcare professionals and work managers to consider when supporting cancer survivors’ work ability [63]. Fatigue has also been seen to be associated with reduced work productivity in oropharyngeal cancer survivors 6 years post radiotherapy [64], further confirming the negative impact of fatigue on work ability. Moreover, healthcare professionals’ attitude towards cancer survivors’ work ability may influence future work ability [65]. Encouragingly, lower fatigue scores were associated with better work ability, highlighting the importance of early, fatigue-focused rehabilitation. These findings should be interpreted with caution, however, as analyses were based on a subgroup predominantly comprising older female breast cancer survivors (58%). The impact of fatigue on males’ work ability requires more research. Male cancer survivors face an even greater risk of reduced employment compared to female [62].

### Methodological considerations

Thorough efforts were made to avoid confounding [66]. By adding a randomly selected reference group from the general population at the time of the cancer survivors’ follow-up, matched for age, sex, and geographic municipality, we aimed to limit bias due to these factors, thus strengthening the results. A strength is the longitudinal design, following up the same individuals who have previously been studied when undergoing radiotherapy. Radiotherapy cohorts are understudied, accounting for only 5% of studied cancer therapy cohorts [67], despite radiotherapy being part of roughly half of all cancer treatment regimens [8]. This longitudinal study included only two assessments—one during radiotherapy and another about 7 years later—limiting conclusions about fatigue in the interim. More frequent assessments would have reduced the risk of capturing temporary fluctuations and better reflected long-term trends. However, the matched cancer-free referents allowed evaluation of the impact of cancer and its treatment on fatigue and functional performance. As in all longitudinal studies with long follow-up periods, it is expected that not all participants remain eligible for follow-up. As anticipated, a substantial proportion had died or met exclusion criteria at follow-up. Although some (up to *n* = 99, unknown *n* still being alive) were lost to follow-up due to an unfortunate administrative failure, we found no differences between them and the responders regarding age, sex or received radiotherapy dose except for having a greater proportion of head and neck cancer (16% versus 6%). Hence this flaw is unlikely to have induced bias, as factors other than cancer type were important for the findings. Regarding potential misrepresentation, the inclusion procedure for eligible cancer survivors including two reminders resulted in a satisfactory [68] response rate of 69%. Among non-responders, the proportion of females was higher than that among responders. Since females are more likely to experience cancer-related fatigue [7, 17], fatigue at follow-up could plausibly have been worse if they had responded. Most female survivors working at follow-up in the present study were in the age-span 45–69 (data not shown). Among female cancer survivors the mean work ability scores were 8.0 (SD 1.4) for those aged < 45, 8.1 (SD 2.1) for those aged 45–69 years, and 8.5 (SD 2.1) for those aged > 69 years with no statistical differences between the age groups (not shown in the result). This suggests that, if similar pattern holds for non-responders, the younger age distribution among them is unlikely to have substantially biased our findings. Additionally, Spearman’s rank correlation analysis among females who were working or studying at follow-up (*n* = 50) revealed a weak and non-significant correlation between age and self-perceived work ability (*r*_s_ = 0.215, *P* = 0.135), further supporting our interpretation. The response rate among the referents was as expected somewhat lower than among the cancer survivors but still satisfactory, 61% [68]. Non-response bias cannot be excluded, as respondents may represent individuals with either more pronounced symptoms or, conversely, particularly good health. However, the referents’ mean EQ-VAS score of 77 aligns with the previously reported mean of 78 for the Swedish general population aged 70–74 years [69].

To minimize misclassification and measurement errors [66], the single-item fatigue measure was developed and validated [19] in accordance with clinimetric methodology [37], in which the target population is actively involved, and their understanding of what fatigue means in daily life is taken into account. The measure does not differentiate between physical, mental, or social dimensions of fatigue; rather, it captures two dimensions of the symptom according to the Theory of Unpleasant Symptoms [51] within a single question. We capture the frequency and the nature of cancer-related fatigue, i.e., an extreme tiredness that is hard to relieve by resting, i.e., fatigue that is a persistent experience. However, the use of this study-specific fatigue measure limits the ability to compare our results with findings form other studies. Although our measure captures overall fatigue, a more comprehensive assessment is needed to distinguish between specific subtypes, such as cognitive fatigue, particularly in relation to work ability. Both fatigue and cognitive complaints have been shown to be negatively associated with work ability [42]. The clinimetric [37] single-item fatigue measure requires additional psychometric evaluation to further establish its psychometric properties. We measured physical and daily activity, and self-perceived work ability, using valid [39–41] and sensitive [26] methods, although objective measures such as accelerometers for measuring physical activity obviously reach higher validity. The daily activity measure [40] seemed to suffer from roof effect; the cancer survivors managed all activities (i.e., *md*) at both time-points. A limitation of the study is that we rely solely on self-reported data, as, for reasons of confidentiality, we do not have access to objective information, such as medical records, regarding cancer status and therapies at follow-up. Consequently, some of the fatigue reported by cancer survivors may be attributable to current cancer. This self-reported data may be subject to validity limitation. However, the aim of the present study was not to investigate the underlying causes of fatigue but rather identify factors that healthcare professionals should be aware of during and long-term after radiotherapy to better support cancer survivors.

To avoid bias due to analytical adjustments [66], we choose GLMM as it is well suited to violations of normality. We analyzed numeric rating scales, i.e., ordinal variables (e.g., fatigue, anxious and depressed mood, pain), as continuous variables. The skewness and kurtosis values of the GLMM’s residuals indicated no substantial deviation from normality. However, more weight should be placed on the direction of the presented associations than on the size of the parameter estimates. The risk of bias and finding statistical differences due to multiple comparisons over time and between cancer survivors and referents seems to be low as we pre-specified just a few important dependent variables (fatigue, daily and physical activity, work ability). Adjustment for multiple testing according to the Holm’s step-down method would not have altered the interpretation of the longitudinal comparisons (adjusted *P*-values ranging from 0.010 to 0.040, not presented in the results). The external validity is strengthened by including male and female cancer survivors, treated for a great variation of cancer types in standard cancer care with few exclusion criteria. Since all participants had the capacity to give informed consent, this limits the generalizability to survivors in very poor condition, or with severe linguistic barriers.

### Implications for practice and research

Fatigue during treatment may serve as an early signal of long-term fatigue independent of concurrent symptoms and depressed mood in cancer survivors, whose experience of fatigue at 7-year follow-up was worse compared to referents. Future studies are warranted to examine whether early identification and supportive strategies may influence later fatigue and its potential implications for work ability in cancer survivors.

## Supplementary Information

Below is the link to the electronic supplementary material.

ESM 1 (DOCX 52.4 KB)

## Data Availability

Data will be made available by the authors upon reasonable request.
