# Supplementary material for: Fatigue, physical and daily activity, and self-perceived work ability in cancer survivors: a long-term follow-up study with a matched non-cancer reference group
Source: J Cancer Surviv. 2026 May 21;20(Suppl 2):2078–95. doi: 10.1007/s11764-026-02041-1 (PMC13744462; doi:10.1007/s11764-026-02041-1)
Supplement: Supplementary file 1 [file 11764_2026_2041_MOESM1_ESM.docx]

Supplemental material

**Fatigue, Physical and Daily Activity, and Self-Perceived Work Ability in Cancer Survivors: A Long-Term Follow-Up Study with a Matched Non-Cancer Reference Group**

**Submitted to: Journal of Cancer Survivorship**

Tödt, K^1^, Engström M^1^, Ekström, M^2^, Heiden M^3^, de Boer A. G. E. M^4^, Efverman A^1^.

^1^Department of Caring Science, Faculty of Health and Occupational Studies, University of Gävle, Sweden, ^2^Respiratory Medicine, Allergology and Palliative Medicine, Department of Clinical Sciences Lund, Lund University, Sweden, 3Occupational Health, Psychology and Sports Sciences, Faculty of Health and Occupational Studies, University of Gävle, ^4^Department of Public and Occupational Health, Amsterdam UMC - University of Amsterdam, Amsterdam, the Netherlands

**Corresponding author:**

Kristina Tödt	[kristina.todt@hig.se](mailto:kristina.todt@hig.se)

Department of Caring Science, Faculty of Health and Occupational Studies

University of Gävle

801 76 Gävle

Sweden

Table S1. Questions in the questionnaire regarding fatigue, daily and physical activity, anxiety and depressed mood, work-related questions

| Questions | Answer options |
| --- | --- |
| **Fatigue** |  |
| Have you felt an extreme tiredness that has been difficult to relieve by resting, during the past week? | All the time  0 1 2 3 4 5 6 7 8 9 10  Never |
| **Daily and physical activity** |  |
| How much of your usual daily activities have you been able to perform during the past week? | 0= I have not managed any of my daily activities  1= I have managed few (less than 1/5) of my daily activities  2= I have managed less than half of my daily activities  3= I have managed approximately half of my daily activities  4= I have managed more than half of my daily activities  5= I have managed most (more than 4/5) of my daily activities  6= I have managed all my daily activities |
| How much time do you spend at a level that makes you short winded, for example, running, fitness class or ball games?^a^ | 0= 0 minutes  1= Less than 30 minutes  2= 31-60 minutes (0,5 – 1 h)  3= 61-90 minutes (1-1,5 h)  4= 91-120 minutes (1,5-2 h)  5= More than 120 minutes (2 h) |
| How much time are you physically active in ways that are not exercise, for example, walks, bicycling, or gardening? Add together all activities lasting at least 10 minutes.^a^ | 0= 0 minutes, no time  1= Less than 30 minutes  2= 31-60 minutes (0,5 – 1 h)  3= 61-90 minutes (1-1,5 h)  4= 91-150 minutes (1,5-2,5 h)  5= 151-300 minutes (2,5-5 h)  6= More than 300 minutes (5 h) |
| **Anxious and depressed mood** |  |
| Have you experienced anxious mood during the past week? | 0 1 2 3 4 5 6 7  Never  All the time |
| Have you experienced depressed mood during the past week? | Never  0 1 2 3 4 5 6 7  All the time |
| **Work-related questions** |  |
| On a scale from 0 to 10 where 0 is the worst job performance anyone could have at your job and 10 is the performance of a top worker, how would you rate your work performance? | Worst performance  Top performance  0 1 2 3 4 5 6 7 8 9 10 |
| We assume that your work ability, when it was at its best, is valued at 10. What score would you give your current work ability? | 0 1 2 3 4 5 6 7 8 9 10  Can´t work at all  My ability to work is at its best |
| Work efficacy: How confident are you in your ability to work?^c^ | Completely confident  Not  confident  0 1 2 3 4 5 6 7 8 9 10 |
| How was your ability to work before your cancer? (cancer survivors only)^b^ | 0 1 2 3 4 5 6 7 8 9 10  No ability/ could not work  Best possible working ability |
| Questions about perceived working life:   - My work is meaningful - I feel good at my work - I have a good balance between work and leisure | 0 1 2 3 4 5 6 7  Agree completely  Don´t agree |
| One question about experienced problems related to work   - Worries about not being able to perform the work | Answer option if not relevant: “Not relevant to me, I have not worked during the past 12 months”  0 1 2 3 4 5 6 7  Agree completely  Don´t agree |
| One question regarded the work situation in relation to the manager:   - I have been given a high degree of flexibility from my supervisor (manager) when it comes to execution of the work | The statement also has an answer option “Not relevant to me”  0 1 2 3 4 5 6 7  Not true at all  Completely true |
| Which actors/persons have you felt supported by regarding being able to continue working or regarding returning to work?   - Manager? - Colleagues /co-workers? - Family and / or other relatives? | Best possible support  0 1 2 3 4 5 6 7  No support |

^a^These questions were answered regarding the past week. ^b^Retrospectively measured at follow-up.
